# Metformin inhibits ovarian granular cell pyroptosis through the miR-670-3p/NOX2/ROS pathway

**DOI:** 10.18632/aging.204745

**Published:** 2023-05-25

**Authors:** Li-Hua Zhou, Hui Zou, Jia-Yuan Hao, Yong Huang, Jia-Nan Zhang, Xiao-Hong Xu, Juan Li

**Affiliations:** 1 The Department of Reproductive Medicine, The Second Affiliated Hospital of Hainan Medical University, Haikou 570100, Hainan, China; 2 Institute of Clinical Medicine, The Second Affiliated Hospital of Hainan Medical University, Haikou 570100, Hainan, China

**Keywords:** metformin, miR-670-3p, NOX2, ROS, pyroptosis

## Abstract

Recent studies have demonstrated that ovarian granular cells (OGCs) pyroptosis is present in the ovaries of polycystic ovary syndrome (PCOS) mice and that NLRP3 activation destroys follicular functions. Metformin has been shown to protect against PCOS by reducing insulin resistance in women, whereas its role in OGC pyroptosis is unknown. This study aimed to investigate the impact of metformin on OGC pyroptosis and the underlying mechanisms. The results showed that treating a human granulosa-like tumor cell line (KGN) with metformin significantly decreased LPS-induced expression of miR-670-3p, NOX2, NLRP3, ASC, cleaved caspase-1, and GSDMD-N. Cellular caspase-1 activity; ROS production; oxidative stress; and the secretion of IL-1β, IL-6, IL-18, and TNF-α were also diminished. These effects were amplified by adding N-acetyl-L-cysteine (NAC), a pharmacological inhibitor of ROS. In contrast, metformin’s anti-pyroptosis and anti-inflammatory effects were robustly ameliorated by NOX2 overexpression in KGN cells. Moreover, bioinformatic analyses, RT-PCR, and Western blotting showed that miR-670-3p could directly bind to the NOX2 (encoded by the CYBB gene in humans) 3’UTR and decrease NOX2 expression. Metformin-induced suppression of NOX2 expression, ROS production, oxidative stress, and pyroptosis was significantly alleviated by transfection with the miR-670-3p inhibitor. These findings suggest that metformin inhibits KGN cell pyroptosis via the miR-670-3p/NOX2/ROS pathway.

## INTRODUCTION

Polycystic ovarian syndrome (PCOS) is a heterogeneous syndrome caused by complex endocrine and metabolic abnormalities in women of childbearing age. It is characterized by persistent anovulation, hyperandrogenemia, and insulin resistance [1]. The main clinical manifestations of PCOS include irregular menstrual cycles, infertility, and hirsutism [2]. The etiology of PCOS remains unclear, but there is increasing evidence that follicular dysfunction is the fundamental reason for PCOS, especially in China [3]. Ovarian granular cells (OGCs) can supply 85% of the nutrients for the growth and development of oocytes, which affects the initiation, development, maturation, and atresia of follicles. In both female patients and animal models, the progression of PCOS is accompanied by a loose arrangement of OGCs, increased cystic follicles, and a thickened thecal cell layer [3, 4]. Moreover, many findings have shown that, compared to healthy controls, the apoptosis rates of OGCs in antral follicles in PCOS are dramatically increased [5, 6], indicating that improving the survival and function of OGCs may be a promising approach for treating PCOS.

Recently, Wang et al. developed a PCOS mouse model by treating female C57BL/6J mice with a 35-d testosterone continuous-release pellet. They found that, compared to the control mice, the expression of pyroptotic factors and proinflammatory cytokines, including NOD-like receptor protein 3 (NLRP3), apoptosis-associated speck-like protein containing a CARD (ASC), cleaved caspase-1, gasdermin D (GSDMD), interleukin-1β (IL-1β), IL-18 and tumor necrosis factor-α (TNF-α), were significantly higher in the ovaries of PCOS mice. In addition, compared to the ovaries of PCOS mice, ovaries from INF39 (an NLRP3-specific inhibitor)-treated mice displayed more preantral and antral follicles and corpus luteum but fewer atypical follicles. In extracted OGCs, overexpression of NLRP3 using nigericin or lentiviral particles significantly induced cell pyroptosis and elevated the expression of Cyp19α1, Cyp11α1, 3β-hydroxysteroid dehydrogenase (3β-HSD), androgen receptor (AR) and follicle-stimulating hormone receptor (FSHR). The activation of NLRP3 also promoted OGC fibrosis. It destroyed follicular functions by elevating the expression of fibrotic factors, such as transforming growth factor-β (TGF-β), connective tissue growth factor (CTGF), α-smooth muscle actin (α-SMA), β-catenin, collagen I, and collagen IV [7]. Thus, suppressing NLRP3 activation, pyroptosis, and inflammation in OGCs can be considered a novel strategy to prevent PCOS.

Since the 1990s, metformin treatment, with or without clomiphene citrate (CC), has significantly lowered testosterone levels and ameliorated insulin resistance in nonobese and obese women with PCOS [8, 9]. A randomized controlled trial (RCT) of 100 PCOS patients showed that the 6-month ovulation rate was 100% with metformin vs. 37% with placebo (*P*<0.001) [10]. Another RCT by Carmina et al. showed that anovulatory women treated with metformin have more ovulatory cycles than those treated with a placebo [11]. Additionally, PCOS patients treated with metformin significantly improved the clinical pregnancy rate [12]. These findings indicate that metformin is highly beneficial to PCOS patients. However, the exact mechanisms of metformin action in developing PCOS remain unknown. This research was designed to explore the effects of metformin on the activation of NLRP3 inflammasomes and pyroptosis in OGCs and the underlying mechanisms, aiming to provide keen insights into the treatment of PCOS.

## MATERIALS AND METHODS

### Materials

The human granulosa-like tumor cell line (KGN) was purchased from EK Bioscience (#CC-Y1716, Shanghai, China). Dulbecco’s modified Eagle’s medium-F12 (DMEM-F12), fetal bovine serum (FBS), and penicillin-streptomycin solution were purchased from Gibco (Life Technologies, Carlsbad, CA, USA). TRIzol™ reagent, the high-capacity cDNA reverse transcription kit, and Lipo3000 transfection reagent were purchased from Thermo Fisher Scientific (Waltham, MA, USA). The SYBR^®^ Premix Ex Taq™ reagent kit was bought from Takara Bio Inc (Shiga, Japan). Metformin, N-acetyl-L-cysteine (NAC), and 3-(4,5)-dimethylthiahiazol-2,5-diphenytetrazolium bromide (MTT) were obtained from Sigma–Aldrich (St. Louis MO, USA). Lactic dehydrogenase (LDH), reactive oxygen species (ROS), caspase-1 activity, and Hoechst 33342/PI staining assay kits were purchased from Beyotime Biotechnology (Shanghai, China). Malondialdehyde (MDA), glutathione (GSH), glutathione peroxidase (GSH-Px), and superoxide dismutase (SOD) were purchased from Nanjing Jiancheng Bioengineering Institute (Nanjing, China). A NOX activity assay kit was purchased from Genmed Pharmaceutical Technology (Shanghai, China). The Dual-Luciferase Reporter Assay System and Renilla psiCHECK2 vector were purchased from Promega (Madison, WI, USA). The QuickChange Site-Directed Mutagenesis kit was purchased from Agilent Technologies (Santa Clara, CA, USA). Small interfering RNAs (siRNAs) used for the knockdown of NOX2 (si-NOX2) and the scrambled control (si-NC), miR-670-3p mimic (miR Mim), mimic control (Mim Con), miR-670-3p inhibitor (miR Inh), and inhibitor control (Inh Con) were obtained from GenePharma Co., Ltd. (Suzhou, China). The lentiviral vector encoding NOX2 (LV-NOX2) and a control empty lentiviral vector (LV-NC) were purchased from OriGene Technologies (Rockville, MD, USA). The human IL-6 ELISA kit (#YIF-LF-EK0260), IL-1β ELISA kit (#K4227-100), and IL-18 ELISA kit (#K4227-100) were purchased from Neobioscience Technology Co., Ltd. (Beijing, China). The primary antibodies used in this study were as follows: NLRP3 (#15101) from Cell Signaling Technology Inc. (CST, Beverly, MA, USA); ASC (#sc-514415) from Santa Cruz Biotechnology (Dallas, TX, USA); NOX2 (#ab80508), Caspase-1 (#ab207802), GSDMD (#ab210070), and GAPDH (#ab181602) from Abcam (Cambridge, MA, USA). Immobilon Western Chemiluminescent HRP Substrate was purchased from Merck company (Darmstadt, Germany).

### Cell culture and transfection

Human KGN cells were cultured in DMEM-F12 containing 10% FBS at 37° C with 5% CO_2_. Before the experiments, cells were cultured in DMEM without antibiotics or FBS for at least 8 h to establish synchronized growth. Before transfection, KGN cells were seeded into a 6-well plate (2×10^5^/plate). Then, the cells were treated with 100 nM miR-670-3p mimic/inhibitor or the corresponding negative controls using Lipo3000 reagent for 48 h. RT-PCR was conducted to assess the transfection efficiency. For NOX2 overexpression, 2.5×10^7^ TU/mL LV-NOX2 or LV-NC was transfected into KGN cells using 8 μL Lipo3000. 48 h later, the cells were harvested for Western blot analyses or other experiments. For NOX2 knockdown, cells were transfected with 100 nM si-NC or si-NOX2 for 48 h, followed by Western blot analyses.

### MTT assay

When the cells were 80% confluent, they were treated with different doses of metformin (0, 5, 10, 20, or 40 μM) for 12 h or 20 μM metformin for various times (0, 6, 12, 24, or 48 h). Then, the cells were incubated with 20 μL of 4 mg/mL MTT solution at 37° C for 4 h. The medium was carefully removed, and 150 μL DMSO was added to each well. Each well’s optical density (OD) value was measured at 490 nm using a Multimode plate reader (PerkinElmer, Inc., Waltham, MA, USA).

### Bioinformatics prediction

The genomic and miR-670-3p sequences were obtained from miRDB, which predicts miRNA targets in various species. TargetScan and miRBase web servers were used to identify miR-670-3p binding sites. The RNAhybrid website evaluated the minimum free energy of hybridization between miR-670-3p and NOX2 (encoded by the CYBB gene in humans).

### Real-time PCR

Total RNA was isolated from cultured cells using TRIzol reagent as described previously [13]. In brief, KGN cells were digested with TRIzol reagent for 5 min at 25° C, and the supernatants were obtained by centrifugation. Then, they were mixed with 50% isopropyl alcohol and centrifuged at 4,000 rpm for 8 min at 4° C. The supernatants were discarded, and the precipitate was mixed with DEPC water to measure the RNA concentration. A high-capacity cDNA reverse transcription kit was used to synthesize complementary DNA. RT–PCR was performed using an SYBR^®^ Premix Ex Taq™ reagent kit on an ABI 7900HT Fast Real-Time PCR System (Applied Biosystems, Foster City, CA, USA). The PCR cycling conditions were as follows: initial denaturation at 95° C for 30 sec, followed by 40 cycles of 95° C for 5 sec, 55° C for 30 sec, and 72° C for 35 sec. The sequences of the primers used are listed in Supplementary Table 1, synthesized by Shanghai Sangon Biotech Co., Ltd. (Shanghai, China).

### Luciferase reporter assay

3’UTR luciferase reporter assays were conducted as described previously [14]. Briefly, the 3’UTR of CYBB, including the wild-type (WT) or mutant-type (Mut) binding sites, was cloned downstream of the Renilla psiCHECK2 vector. Mutations in the predicted binding sites for CYBB were generated using a QuickChange Site-Directed Mutagenesis kit. The 293T cells were cotransfected with 100 nM miR-670-3p mimic/mimic control or miR-670-3p inhibitor/inhibitor control, 200 ng CYBB 3’UTR-WT reporter construct, and 200 ng CYBB 3’UTR-Mut reporter construct using Lipo3000. A dual-luciferase reporter assay system was performed to determine the luciferase activity at 48 h post-transfection. Data were expressed as fold changes (firefly luciferase activity/Renilla luciferase activity).

### Protein extraction and Western blot analyses

Whole-cell extracts of human KGN cells were prepared using a mixture of RIPA buffer and PMSF at a ratio of 94:6. Proteins were isolated by centrifugation, and the BCA protein assay measured the concentrations. Subsequently, the proteins were separated by SDS-PAGE (10% gels, 20 μg protein per lane) and then electrically transferred onto PVDF membranes. The membranes were blocked in 5% nonfat dry milk and 2.5% BSA for 1 h at room temperature and immunoblotted overnight at 4° C with primary antibodies. All primary antibodies were used at a 1:1000 dilution in Tris-buffered saline plus Tween-20 (TBS-T) containing 5% BSA. Afterward, they were incubated with a secondary antibody (1:5000) conjugated to peroxidase for 2 h at room temperature. The targeted proteins were visualized using the Immobilon Western Chemiluminescent HRP Substrate, and the Image J software was used to quantify protein band densities.

### Measurement of LDH release

When the cells reached 80% confluence, they were cultured without serum or antibiotics for 8 h and pretreated with lipopolysaccharide (LPS), metformin, or NAC or transfected with miR-670-3p mimic/inhibitor or LV-NOX2. Then, the treated cells were centrifuged at 400 rpm for 5 min, and the supernatants were collected. The supernatant from each sample (120 μL) was subsequently transferred to a new 96-well plate and mixed with a 60 μL reaction mixture (20 μL lactate, 20 μL INT, and 20 μL diaphorase) for 30 min at room temperature. Finally, the absorbance was detected at 450 nm on a Multimode plate reader.

### Detection of caspase-1 activity

Cells were treated or transfected as mentioned previously and lysed with lysis buffer. The protein concentrations were detected using a BCA assay kit. Then, triplicate aliquots of each sample (10 μL) were incubated with 2 mM Ac-YVAD-pNA (10 μL) in 96-well microplates at 37° C for 2 h, and caspase-1 activity in individual wells was evaluated based on the absorbance at 405 nm using a Multimode plate reader [15].

### Hoechst 33342 and propidium iodide (PI) double staining

KGN cells were plated in replicates in 6-well plates at 1×10^6^ cells/well density. Following treatment or transfection, cells were trypsinized and collected via centrifugation at 3000 rpm for 5 min at 37° C. Subsequently, they were resuspended and incubated with 5 μL Hoechst 33342 (1 mg/mL) and 5 μL PI solution (5 mg/mL) for 15 min at 37° C. A Nikon TS2R-FL was employed to excite fluorescence and take standard pictures.

### ELISA

After treatment, KGN cells were harvested and centrifuged at 400 rpm for 10 min. The cell culture supernatants were then added to 96-well plates. Subsequently, the levels of the inflammatory factors IL-1β, IL-6, IL-18, and TNF-α in the culture media were measured using ELISA kits. The OD value of each well at 450 nm was monitored per the kit instructions.

### Evaluation of ROS production, NOX activity, and oxidative stress

Cells were treated with LPS, metformin, or NAC; or transfected with miR-670-3p mimic/inhibitor or LV-NOX2. Then, they were incubated with 10 μM DCFH-DAfor 20 min at 37° C. The dichlorofluorescein (DCF) fluorescence distribution was detected using a fluoro-spectrometer (NanoDrop™ 3300) at an excitation wavelength of 488 nm and an emission wavelength of 535 nm. Moreover, cells were centrifuged, and supernatants were collected for the detection of NOX activity and the production of (anti-)oxidant enzymes, including MDA, SOD, GSH, and GSH-px, using commercial kits.

### Statistical analysis

All data are expressed as the mean ± standard deviation (S.D.) from at least three independent experiments. All statistical analyses among groups were performed by one-way ANOVA followed by Tukey’s multiple comparison test, using GraphPad Prism 9.5 software (San Fiego, CA, USA). A value of *P* < 0.05 was considered statistical significance.

## RESULTS

### Metformin inhibits LPS-induced cell pyroptosis and reduces the secretion of inflammatory factors in human KGN cells

**Figure 1:**
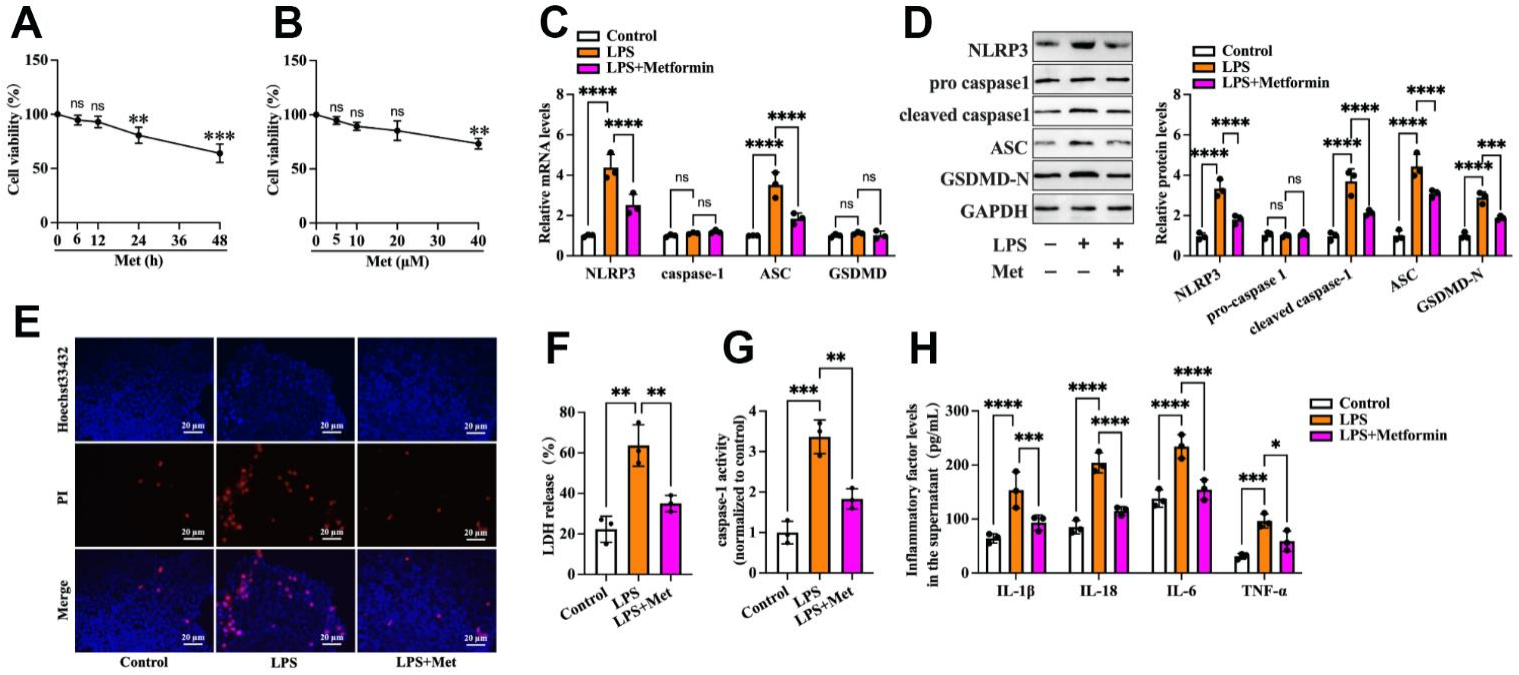
**Metformin inhibits LPS-induced KGN cell pyroptosis.** (**A**, **B**) Cells were incubated with different concentrations of metformin (0, 5, 10, 20, 40 μM) for 12 h or 20 μM metformin for various periods (0, 6, 12, 24, 48 h). Then, an MTT assay was conducted to assess cell viability. (**C**–**H**) Cells were pretreated with or without 10 μM LPS for 24 h and then incubated with 20 μM metformin for another 12 h; (**C**) Quantification of mRNA expression of NLRP3, caspase-1, ASC, and GSDMD using RT-PCR; (**D**) Western blot analyses of NLRP3, pro-caspase-1, cleaved-caspase-1, ASC, and GSDMD-N; (**E**) Representative micrographs of PI staining (red) and Hoechst 33342 staining (blue); (**F**) Levels of LDH released into the cell culture medium from membrane pores were examined using an LDH cytotoxicity detection kit; (**G**) Caspase-1 activity was assessed using a colorimetric caspase-1 activity assay kit; (**H**) The levels of IL-1β, IL-18, IL-6, and TNF-α in the cell culture supernatant were determined by ELISA. Data are represented as the means ± SD from three independent experiments. *P < 0.05, **P < 0.01, ***P < 0.001, ****P < 0.0001, ns. not significant.

First, we detected the effects of metformin on the viability of human KGN cells. Cells were treated with various concentrations of metformin (0, 5, 10, 20, and 40 μM) for 12 h or 20 μM metformin for different durations (0, 6, 12, 24, and 48 h). The MTT assay showed that treatment with 20 μM metformin for 12 h had no significant cytotoxic effects (Figure 1A, 1B). Thus, 20 μM metformin for 12 h was chosen for the following experiments. To ascertain whether metformin affects KGN cell pyroptosis, the cells were divided into three groups: (1) the control group, (2) the LPS group, and (3) the LPS + metformin group. As shown in Figure 1, compared with the control group, exposure of KGN cells to LPS resulted in a significant increase in the expression of pyroptosis-related factors (NLRP3, ASC, cleaved caspase-1 and GSDMD-N), LDH release, and caspase-1 activity and a decrease in plasma membrane integrity (Figure 1C–1G). In addition, the secretion of proinflammatory factors, including IL-1β, IL-6, IL-18, and TNF-α, was also elevated (Figure 1H). Nevertheless, metformin treatment markedly compromised these effects (Figure 1C–1H), indicating that metformin could suppress LPS-induced cell pyroptosis and inflammation in human KGN cells.

### The NOX2-ROS pathway is involved in metformin-induced repression of cell pyroptosis and inflammation in human KGN cells

**Figure 2:**
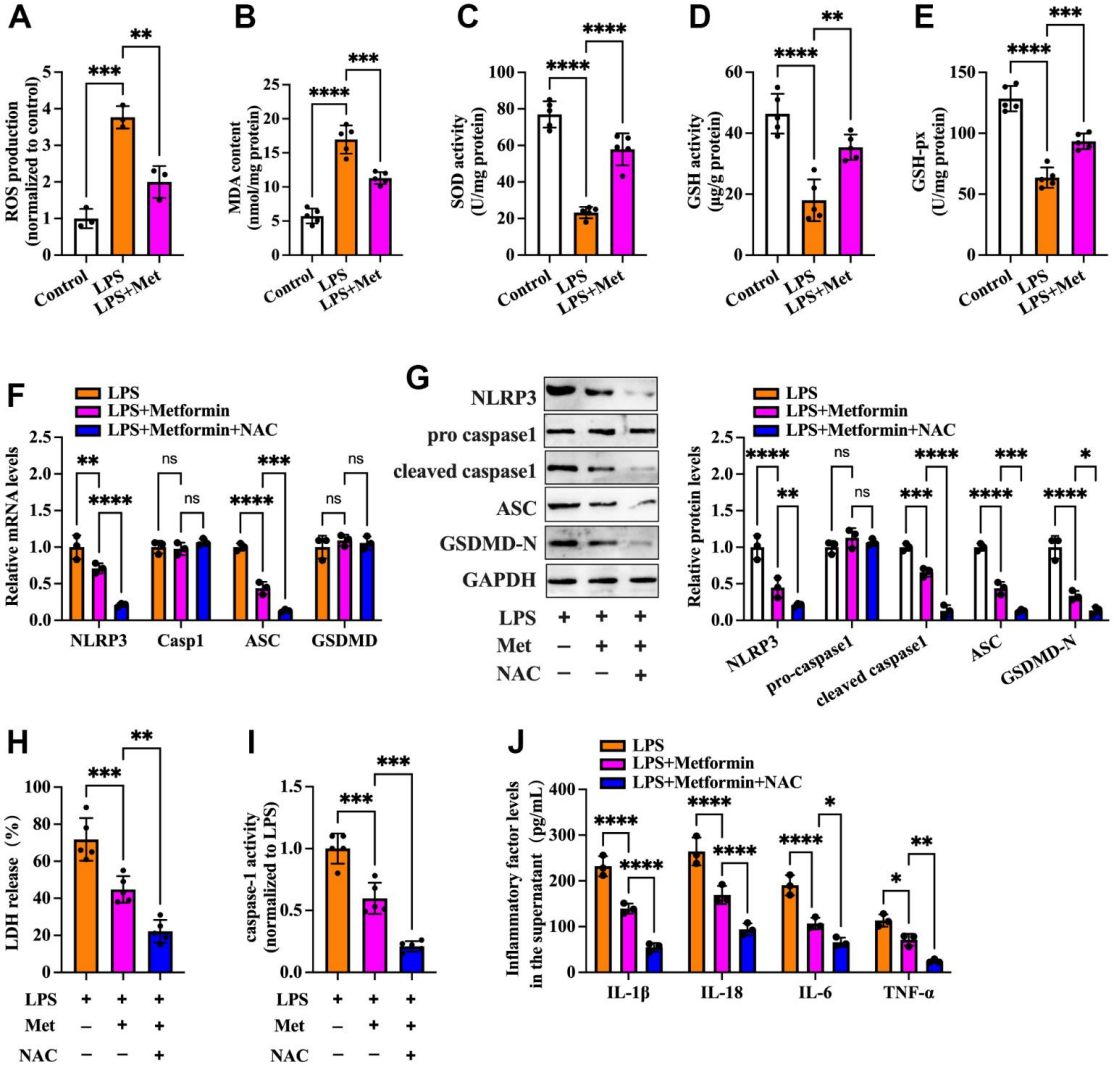
**Metformin inhibits inflammation and KGN cell pyroptosis by decreasing ROS production and oxidative stress.** (**A**–**E**) Cells were pretreated with or without 10 μM LPS for 24 h and then incubated with 20 μM metformin for another 12 h. Intracellular levels of ROS, MDA, SOD, GSH, and GSH-px were measured using respective commercial kits; (**F**–**J**) Cells were pretreated with 10 μM LPS for 24 h and then incubated with 20 μM metformin and 10 mM NAC for another 12 h; (**F**) RT-PCR detected the mRNA levels of NLRP3, caspase-1, ASC, and GSDMD; (**G**) The protein levels of NLRP3, pro-caspase-1, cleaved-caspase-1, ASC and GSDMD-N were detected by Western blot; (**H**) Levels of LDH in the cell culture medium were examined using an LDH cytotoxicity detection kit; (**I**) Caspase-1 activity was assessed using a colorimetric caspase-1 activity assay kit; (**J**) The levels of IL-1β, IL-18, IL-6, and TNF-α in the cell culture supernatant were determined by ELISA. Data are represented as the means ± SD from three independent experiments. *P < 0.05, **P < 0.01, ***P < 0.001, ****P < 0.0001, ns. not significant.

ROS are derived from molecular oxygen and are formed by redox reactions or electronic excitation. Oxidative stress, a phenomenon caused by an imbalance between radicals and antioxidant defense, is a primary pathophysiological mechanism in various human diseases [16]. It has been reported that ROS accumulation and increased oxidative stress are present in OGCs of PCOS patients [17, 18]. We next explored whether metformin influences cell pyroptosis and inflammation via antioxidant action. As shown in Figure 2A–2E, compared with the controls, treatment with LPS led to elevated ROS production and oxidative stress in KGN cells, as evidenced by increased levels of ROS and MDA and decreased levels of SOD, GSH, and GSH-px, whereas metformin treatment significantly repressed LPS-induced ROS production and oxidative stress. Notably, the inhibitory effects of metformin on the expression of pyroptosis-related factors (NLRP3, ASC, cleaved caspase-1, and GSDMD-N), LDH release, caspase-1 activity, and secretion of proinflammatory cytokines were more apparent after treatment with NAC, a ROS scavenger (Figure 2F–2J). These findings indicated that metformin suppresses pyroptosis and inflammation in human KGN cells by decreasing ROS production and oxidative stress.

**Figure 3:**
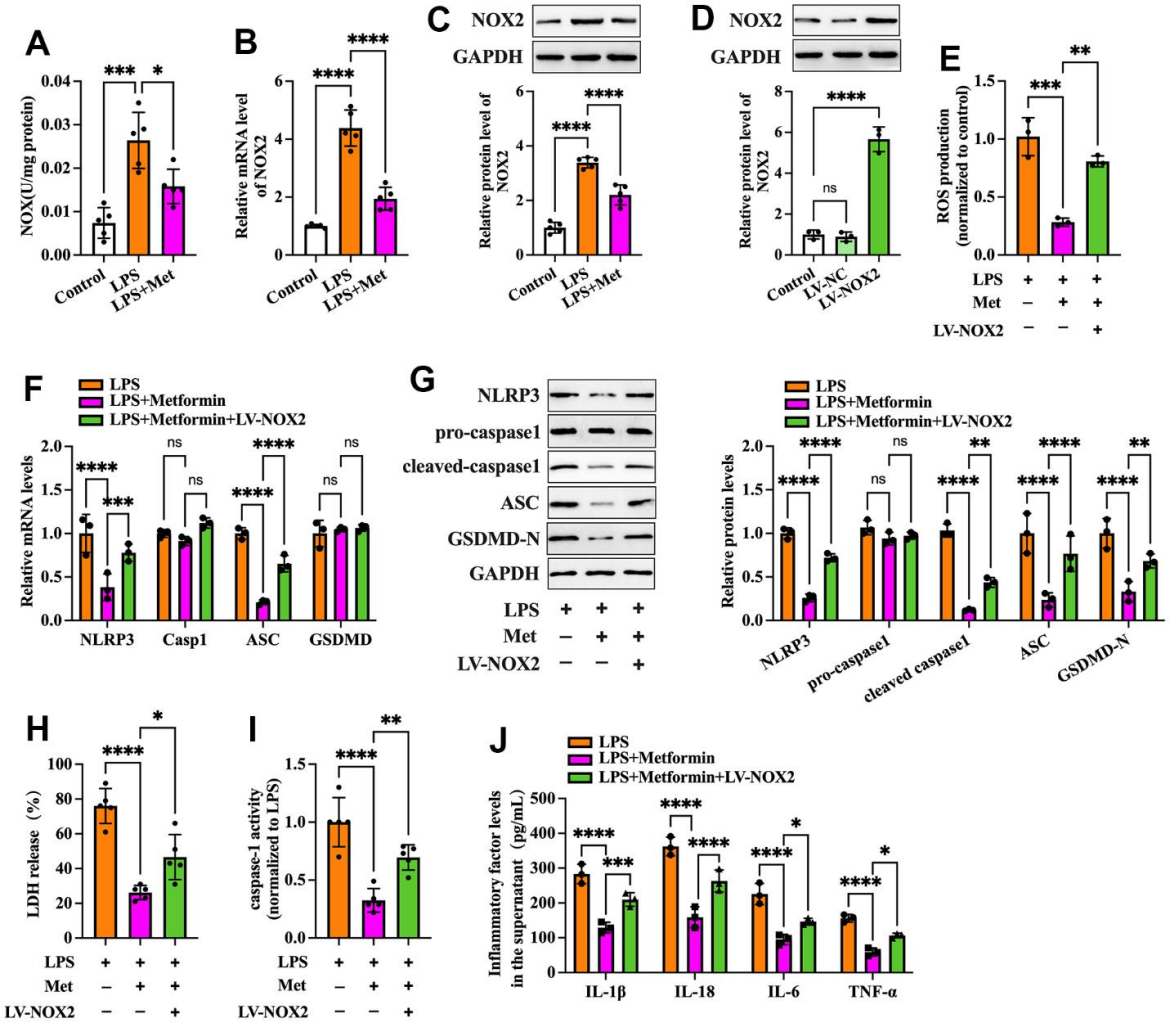
**Effects of NOX2 overexpression on the inhibitory effects of metformin on inflammation and KGN cell pyroptosis.** (**A**–**C**) Cells were pretreated with or without 10 μM LPS for 24 h and then incubated with 20 μM metformin for another 12 h. (**A**) NOX activity was evaluated using a commercial NOX detection kit; (**B**) Quantification of NOX2 mRNA expression using RT-PCR; (**C**) Analysis of NOX2 protein levels using Western blotting; (**D**) Cells were transduced with PBS, LV-NC, or LV-NOX2 for 48 h. The transfection efficiency was evaluated using Western blot analysis; (**E**–**J**) Cells were pretreated with 10 μM LPS for 24 h or transfected with LV-NOX2 for 48 h and then incubated with 20 μM metformin for another 12 h; (**E**) Intracellular levels of ROS were measured using commercial kits; (**F**) The mRNA levels of NLRP3, ASC, caspase-1 and GSDMD were detected by RT-PCR; (**G**) The protein levels of NLRP3, pro-caspase-1, cleaved-caspase-1, ASC and GSDMD-N were detected by Western blot; (**H**) Levels of LDH in the cell culture medium were examined using an LDH cytotoxicity detection kit; (**I**) Caspase-1 activity was assessed using a colorimetric caspase-1 activity assay kit; (**J**) The levels of IL-1β, IL-18, IL-6, and TNF-α in the cell culture supernatant were determined by ELISA. Data are represented as the means ± SD from three independent experiments. *P < 0.05, **P < 0.01, ***P < 0.001, ****P < 0.0001, ns. not significant.

**Figure 4:**
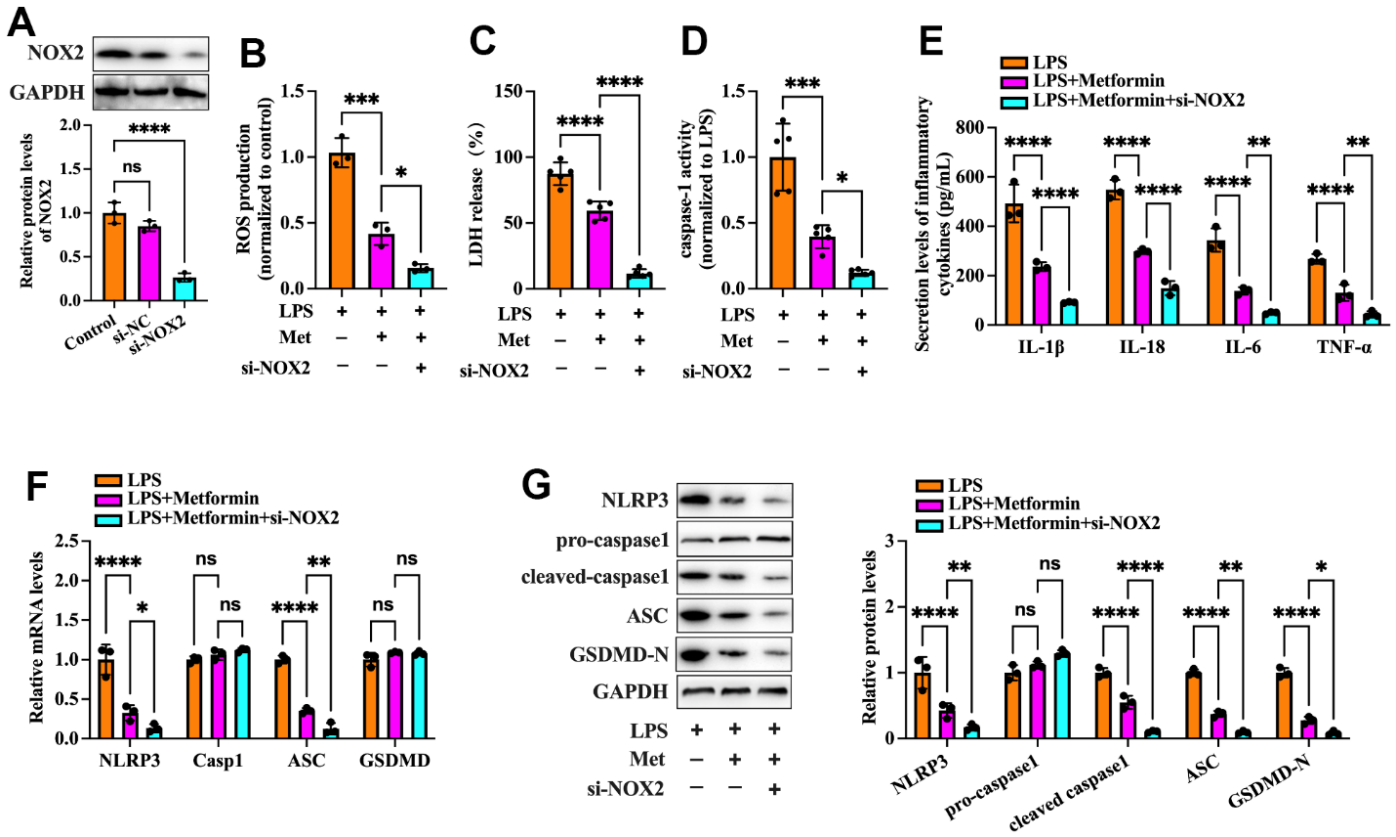
**Effects of NOX2 down-regulation on the inhibitory effects of metformin on inflammation and KGN cell pyroptosis.** (**A**) Cells were transduced with PBS, 100 nM si-NC, or si-NOX2 for 48 h. The transfection efficiency was evaluated using Western blot analysis; (**B**–**G**) Cells were pretreated with 10 μM LPS for 24 h or transfected with si-NOX2 for 48 h and then incubated with 20 μM metformin for another 12 h; (**B**) Intracellular levels of ROS were measured using commercial kits; (**C**) Levels of LDH in the cell culture medium were examined using an LDH cytotoxicity detection kit; (**D**) Caspase-1 activity was assessed using a colorimetric caspase-1 activity assay kit; (**E**) The levels of IL-1β, IL-18, IL-6, and TNF-α in the cell culture supernatant were determined by ELISA. (**F**) RT-PCR detected the mRNA levels of NLRP3, ASC, caspase-1, and GSDMD; (**G**) The protein levels of NLRP3, pro-caspase-1, cleaved-caspase-1, ASC, and GSDMD-N were detected by Western blot; Data are represented as the means ± SD from three independent experiments. *P < 0.05, **P < 0.01, ***P < 0.001, ****P < 0.0001, ns. not significant.

Nicotinamide adenine dinucleotide phosphate (NADPH) oxidases have been identified as one of the critical sources of ROS in many cells. NOX2 NADPH oxidase has been demonstrated to be involved in the ROS production mechanism in isolated GCs of PCOS patients [19]. We further ascertained whether metformin diminishes ROS-mediated pyroptosis and inflammation by inhibiting the expression of NOX2 in KGN cells. As shown in Figure 3A–3C, exposure to LPS significantly increased NOX activity and NOX2 expression compared to the controls, while metformin treatment abolished these effects. Next, we transfected KGN cells with LV-NC or LV-NOX2 for 48 h to overexpress NOX2. A more significant than 4.03-fold elevation of NOX2 levels was observed after transfection with LV-NOX2 (Figure 3D). Furthermore, the suppressive effects of metformin on ROS production, cell pyroptosis and secretion of proinflammatory factors were robustly compromised upon NOX2 overexpression (Figure 3E–3J). In contrast, transfection of NOX2 siRNA presented the opposite effects (Figure 4). Altogether, these data support the concept that the NOX2-ROS pathway is involved in metformin-induced repression of cell pyroptosis and inflammation in human KGN cells.

### Identification of NOX2 as a direct target of miR-670-3p

**Figure 5:**
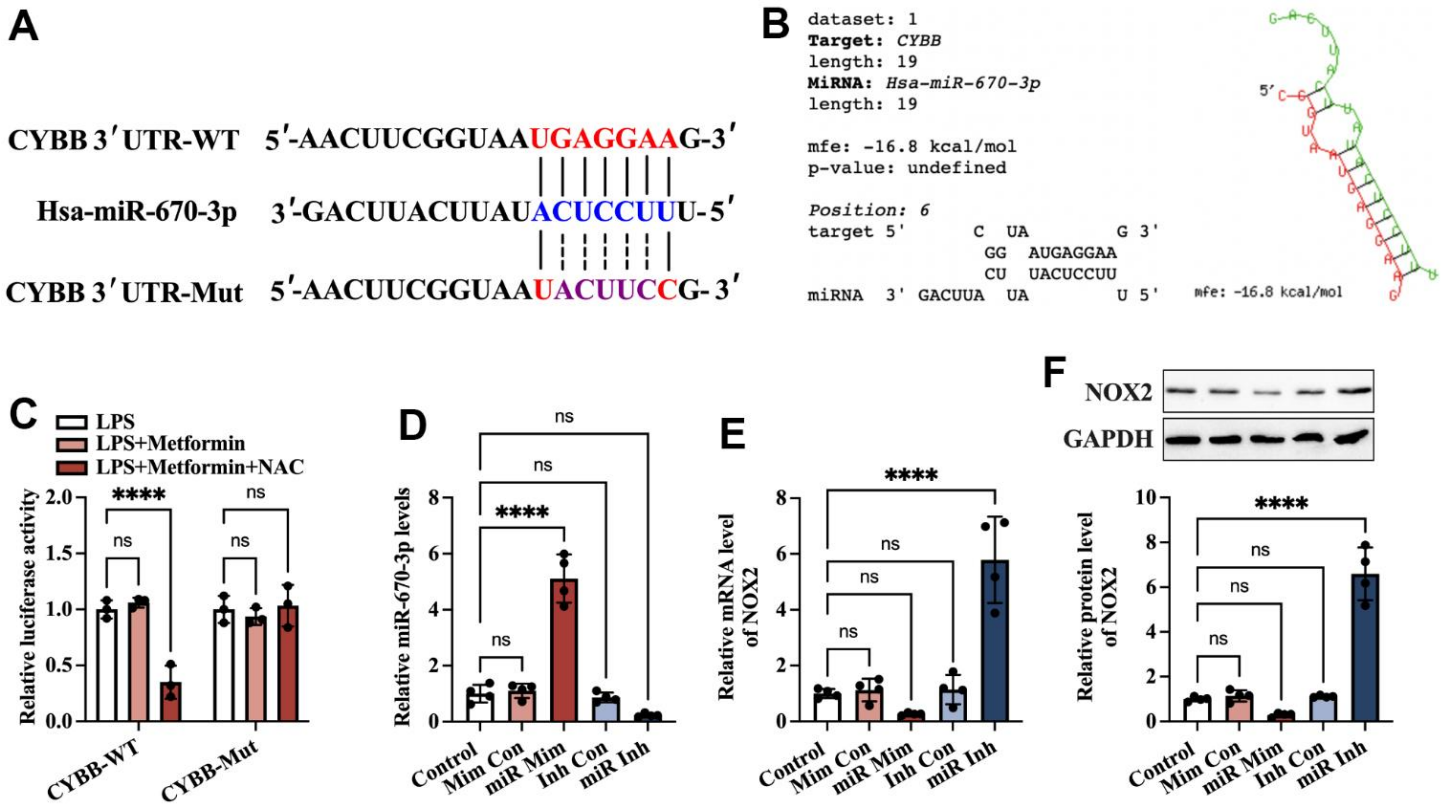
**Identification of CYBB as a direct target of miR-670-3p.** (**A**) Schematic diagram showing the predicted miR-670-3p binding site in the human CYBB 3’UTR and corresponding mutation sites. (**B**) Calculation of the minimum free energy hybridization of miR-670-3p and CYBB 3*’* UTR using RNAhybrid tools; (**C**) The luciferase reporter plasmids (CYBB-WT and CYBB-Mut) were cotransfected into 293T cells with miR-670-3p mimic or its negative control. Forty-eight hours later, the luciferase activity was detected; (**D**–**F**) KGN cells were transfected with miR-670-3p mimic/inhibitor or their negative controls for 48 h; (**D**) RT-PCR analysis of miR-670-3p expression; (**E**, **F**) The mRNA and protein levels of NOX2 were determined by RT-PCR and Western blot, respectively. Data are represented as the means ± SD from three independent experiments. ****P < 0.0001, ns. not significant.

Accumulating evidence has shown that miRNAs regulate NOX2 (encoded by the CYBB gene in humans) at the transcriptional level [20, 21]. To identify potential miRNAs that can target CYBB, bioinformatics analyses were performed using the miRDB and TargetScan websites. The results showed that there was a conserved putative binding site between miR-670-3p and the CYBB 3’ UTR (Figure 5A). In addition, RNAhybrid results showed that the hybridization energy between miR-670-3p and the CYBB 3’ UTR is approximately -16.8 kcal/mol, suggesting that miR-670-3p can stably bind to the CYBB 3’ UTR (Figure 5B). To explore further the target prediction algorithms, a luciferase reporter plasmid containing the wild-type (CYBB-WT) or mutant (CYBB-Mut) miR-670-3p binding site was constructed (Figure 5A). 293T cells were transfected with these plasmids with the miR-670-3p mimic or mimic control. According to the luciferase reporter assay results, transfection of CYBB-WT and miR-670-3p mimic notably decreased the luciferase activity. In contrast, this effect disappeared in the CYBB-Mut group (Figure 5C). KGN cells were transfected with miR-670-3p mimic/inhibitor and their corresponding controls. Compared to the control group, treatment with the miR-670-3p mimic increased miR-670-3p levels by 4.11-fold, while its inhibitor decreased miR-670-3p levels by 76.8% (Figure 5D), indicating a high transfection efficiency. Moreover, transfection with miR-670-3p mimic downregulated the mRNA and protein levels of NOX2, while the opposite effect was observed in the miR-670-3p inhibitor group (Figure 5E, 5F). These results suggested that miR-670-3p can directly combine with the CYBB 3’ UTR and suppress NOX2 expression.

### MiR-670-3p is required for metformin-induced inhibition of the NOX2-ROS pathway and KGN cell pyroptosis

**Figure 6:**
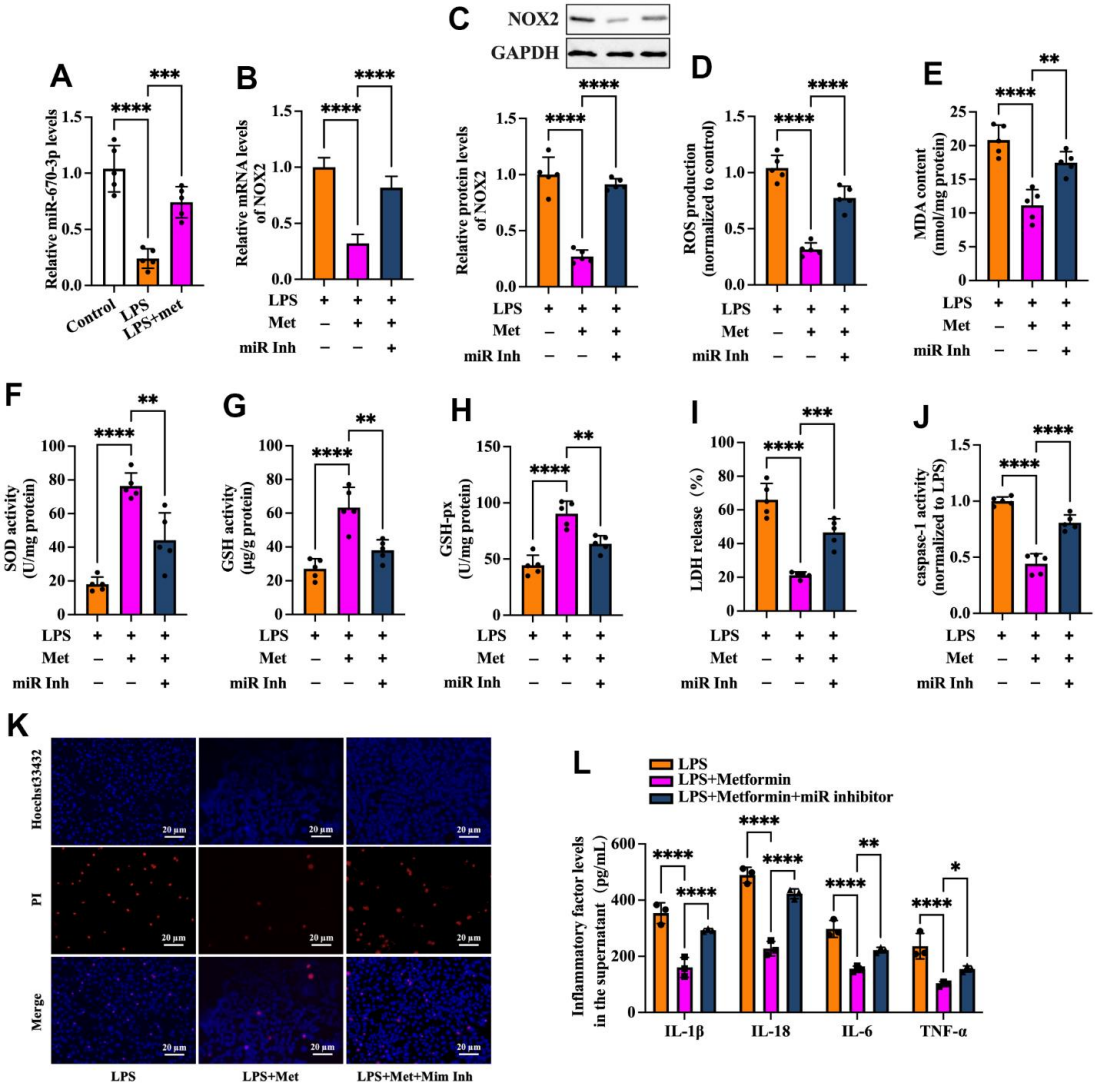
**Metformin inhibits ROS production, oxidative stress, and KGN cell pyroptosis by increasing miR-670-3p.** (**A**) Cells were pretreated with or without 10 μM LPS for 24 h and then incubated with 20 μM metformin for another 12 h. miR-670-3p levels were detected using RT-PCR; (**B**–**L**) Cells were pretreated with 10 μM LPS for 24 h or transfected with miR-670-3p inhibitor for 48 h and then incubated with 20 μM metformin for another 12 h; (**B**) Quantification of mRNA expression of NOX2 using RT-PCR; (**C**) Analysis of NOX2 protein level using Western blot; (**D**–**H**) Intracellular levels of ROS, MDA, SOD, GSH, and GSH-px were measured using commercial kits; (**I**) Levels of LDH in the cell culture medium were examined using an LDH cytotoxicity detection kit; (**J**) Caspase-1 activity was assessed using a colorimetric caspase-1 activity assay kit; (**K**) Representative micrographs of PI staining (Red) and Hoechst 33342 staining (Blue); (**L**) The levels of IL-1β, IL-18, IL-6, and TNF-α in the cell culture supernatant were determined by ELISA. Data are represented as the means ± SD from three independent experiments. **P < 0.01, ***P < 0.001, ****P < 0.0001. Scale bar=20 μm.

Based on the above studies, we speculated that miR-670-3p likely mediates metformin-induced suppression of the NOX2-ROS pathway and cell pyroptosis. To verify this possibility, RT-PCR was used to detect miR-670-3p expression in KGN cells treated with LPS or metformin. As shown in Figure 6A, exposure of KGN cells to LPS markedly decreased miR-670-3p levels, while metformin significantly compromised this effect. Subsequently, KGN cells were transfected with miR-670-3p inhibitor before metformin treatment. As shown in Figure 6B–6H, the negative effects of metformin on NOX2 expression, ROS production, and oxidative stress were primarily reversed by miR-670-3p inhibitor transfection. Furthermore, metformin-induced promotion of plasma membrane integrity and downregulation of LDH release, caspase-1 activity, and secretion of proinflammatory factors were also compromised by miR-670-3p suppression (Figure 6I–6L). These observations revealed that metformin inhibits the NOX2-ROS pathway, pyroptosis, and inflammation by upregulating miR-670-3p in KGN cells.

## DISCUSSION

The pathogenesis of PCOS may involve a complex group of genetic and environmental factors, including chronic inflammatory reactions, obesity, insulin resistance, and prenatal androgen excess [22]. It is reported that compared to normal mice, plasma concentrations of LPS, IL-6, TNF-α, and IL-17 were significantly increased in PCOS mice. At the same time, metformin treatment decreased the abnormally elevated levels of these factors, suggesting that metformin may prevent PCOS by attenuating LPS-induced endotoxemia [23]. In addition, this remedy can also decrease the hyperreactivity of platelets in PCOS patients by maintaining mitochondrial integrity [24]. Most recently, Huang et al. reported that, in PCOS mice, increased levels of LPS and interferon (IFN)-γ in plasma could induce macrophage pyroptosis in the ovary, while GSDMD knockout (GSDMD^-/-^) mice showed markedly ameliorated macrophage pyroptosis and PCOS development. Mechanistically, macrophage pyroptosis disrupted estrogen synthesis and promoted OGC apoptosis. They further identified that metformin could remarkably reduce serum IFN-γ levels and suppress macrophage pyroptosis in ovaries, thereby ameliorating PCOS [25]. These findings indicated that preventing pyroptosis in ovaries may be regarded as a potential therapeutic strategy for PCOS. However, the exact role of metformin in the pyroptosis of OGCs and the underlying mechanisms have not been investigated thus far. We established a KGN cell pyroptosis model in the present study using LPS. We found that treatment with metformin significantly decreased the expression of pyroptosis-related factors, LDH release and caspase-1 activity, and secretion of proinflammatory cytokines and increased plasma membrane integrity (Figure 1), demonstrating that metformin could suppress pyroptosis and inflammation in human KGN cells.

NOX complexes are a family of transmembrane electron-carrying enzymes responsible for producing ROS, which play a crucial role in autoimmunity and host defense against invading pathogens [26]. Pollock et al. observed that hypochlorous acid (HOCl), a category of ROS with potent antibacterial activity, was not generated in NOX2^-/-^ mice [27]. ROS are bridges linking oxidative stress and NLRP3 inflammasome activation, which drives pyroptosis and promotes tissue inflammation [28]. Zhou et al. found that treatment of melanoma A375 cells with iron and carbonyl cyanide m-chlorophenyl hydrazone (CCCP, an oxidative stress activator) significantly boosted ROS levels, resulting in enhancement of GSDME cleavage, LDH release, and pyroptotic cell death. Moreover, the addition of the ROS inhibitor NAC markedly attenuated the CCCP/iron-induced ROS elevation and cell pyroptosis, indicating that melanoma cell pyroptosis can be induced in a ROS-dependent manner [29]. In addition, Li et al. reported that stimulating cardiomyocytes with LPS amplified intracellular ROS production, which further induced NLRP3 translocation from the nucleus to the cytoplasm. Cytoplasmic NLRP3 directly binds to thioredoxin-interacting protein (TXNIP) and forms an inflammasome, ultimately triggering pyroptosis and exacerbating inflammation in cardiomyocytes [30]. Our data showed that metformin treatment downregulated NOX2 expression and ROS production in KGN cells. Moreover, the inhibitory effects of metformin on ROS production, cell pyroptosis, and inflammation were robustly abrogated by NOX2 overexpression and amplified by ROS inhibition (Figures 2, 3). These observations indicated that metformin ameliorates KGN cell pyroptosis and inflammation by inhibiting the NOX2-ROS pathway.

Evidence has shown that metformin exerts cytoprotective and anti-inflammatory effects by regulating miRNA expression. Xu et al. found that metformin could increase miR-143-3p levels, which target the TGF-β1 3’UTR, decrease TGF-β1 expression and the secretion of TNF-α and IL-6, leading to inhibition of inflammation and proliferation in mesangial cells [31]. In LPS-stimulated RAW264.7 macrophages, metformin treatment significantly ameliorated inflammation, increased the levels of Dicer (a vital enzyme of miRNA biogenesis), and upregulated 83% of miRNAs. Among these miRNAs, the addition of miR-34a-5p and miR-125b-5p inhibitors abolished the suppressive action of metformin on the production of IL-6 and TNF-α [32]. In addition, Docrat et al. identified metformin as a potent neuroprotective factor through the downregulation of miR-141, which enhanced PP2A levels and further inhibited the gene expression of NF-κB, NLRP3, caspase-1, IL-1β, IL-18 and TNF-α [33]. As a pleiotropic miRNA, miR-670-3p is on human chromosome 11p11.2 and closely correlates with cell viability. It has been reported that miR-670-3p can inhibit cell apoptosis and promote lung adenocarcinoma cells’ proliferative and migratory abilities by targeting UHRF1BP1 [34]. Another study found that microinjection of miR-670-3p mimic into mouse embryos caused a significant decrease in cleavage and blastula rates. Specifically, miR-670-3p can target the Igf2bp1 gene and reduce its expression and N^6^-methylation of adenosine (m^6^A) levels, further inducing cell apoptosis in mouse embryos [35]. In the present study, we identified NOX2 as a direct target of miR-670-3p, as evidenced by bioinformatics prediction and luciferase reporter assays (Figure 5). Treatment with metformin resulted in upregulation of miR-670-3p levels in pyroptotic KGN cells. Furthermore, transfection of the miR-670-3p inhibitor notably compromised the suppressive effects of metformin on NOX2 expression, ROS production, oxidative stress, cell pyroptosis, and inflammation (Figure 6), suggesting that metformin inhibits KGN cell pyroptosis and inflammation via the miR-670-3p/NOX2/ROS pathway. Nevertheless, it remains to be determined whether metformin directly or indirectly influences the expression of miR-670-3p.

**Figure 7:**
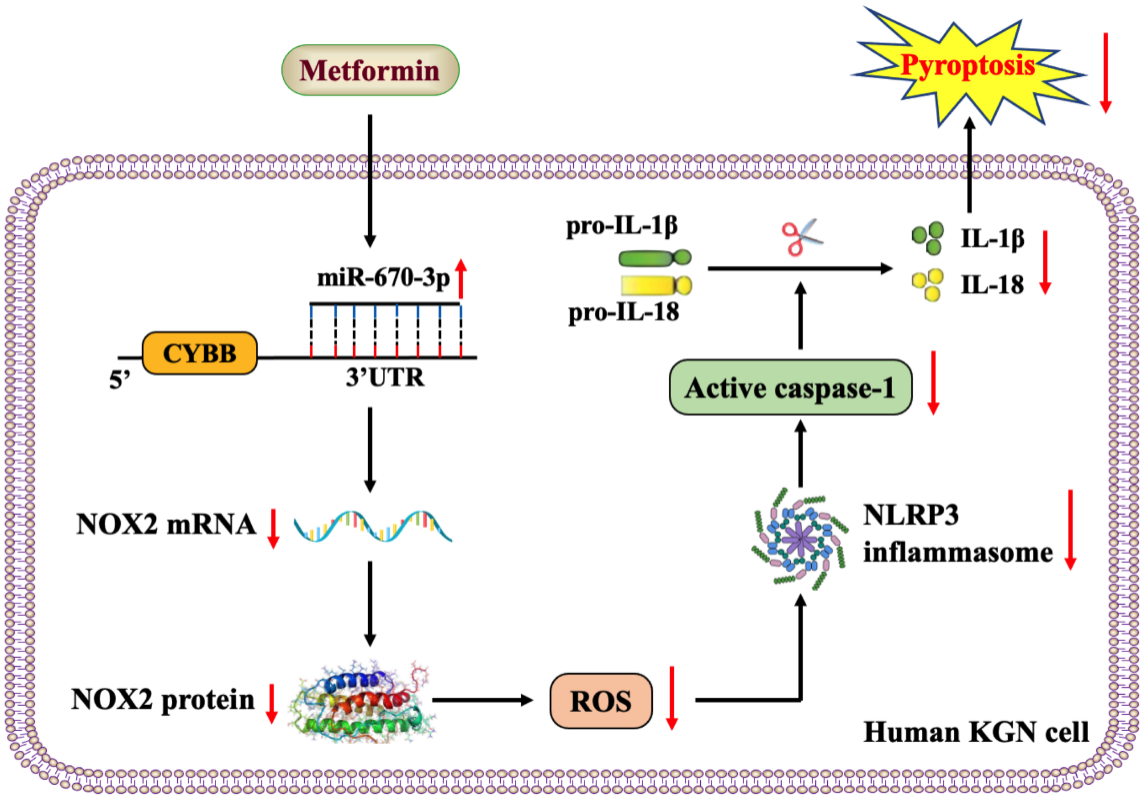
**Schematic overview of the anti-pyroptotic action of metformin in KGN cells.** Metformin increases the levels of miR-670-3p, which in turn targets the CYBB 3’UTR and downregulates NOX2 expression, reduces ROS production, inhibits the activation of the NLRP3 inflammasome and ameliorates KGN cell pyroptosis.

Taken together, the results of our study revealed that metformin elevates miR-670-3p levels and then decreases the transcription of the NOX2 gene, thereby reducing ROS production and activation of the NLRP3 inflammasome and ultimately ameliorating pyroptosis and inflammation in human KGN cells (Figure 7). miR-670-3p and NOX2 may be novel potential therapeutic targets for developing PCOS, which extend the understanding of metformin’s biological functions in gynecological diseases.

## Supplementary Material

Supplementary Table 1
