# Supplementary material for: Metformin inhibits ovarian granular cell pyroptosis through the miR-670-3p/NOX2/ROS pathway
Source: Aging (Albany NY). 2023 May 25;15(10):4429–43. doi: 10.18632/aging.204745 (PMC10258021; doi:10.18632/aging.204745)
Supplement: Supplementary Table 1 [file aging-15-204745-s001.pdf]

SUPPLEMENTARY TABLE

Supplementary Table 1. The primer sequences used in RT-PCR.

miR-670-3p	Forward, 5’-CTGATCGTGAGGAGAGTGT-3’;<br>Reverse, 5’-GGTCTTCGACATCGGGGCGG-3’
NLRP3	Forward, 5’-CAACCTCACGTCACACTGCT-3’;<br>Reverse, 5’-TTTCAGACAACCCCAGGTTC-3’
ASC	Forward, 5’-CTGACGGATGAGCAGTACCA-3’;<br>Reverse, 5’-CAGGATGATTTGGTGGGATT-3’
Caspase-1	Forward, 5’-TGTAATGAAGACTGCTACCTGGC-3’;<br>Reverse, 5’-CCTTGTTTCTGAGGATGAAGGATGT-3’
GSDMD	Forward, 5’-ATGGATGGGCAGATACAGGG-3’;<br>Reverse, 5’-TGCTGCAGGACTTTGTGTTC-3’
NOX2	Forward, 5’-ACAAGGTTTATGACGATGAGCC-3’;<br>Reverse, 5’-TTGAGCAACACGCACTGGAA-3’
U6	Forward, 5’-CGCTTCGGCAGCACATATAC-3’;<br>Reverse, 5’-TTCACGAATTTGCGTGTCAT-3’
GAPDH	Forward, 5’-GGAGCGAGATCCCTCCAAAAT-3’;<br>Reverse, 5’-GGCTGTTGTCATACTTCTCATGG-3’
